# Notes and Notices

**Published:** 1888-05

**Authors:** 


### NOTES AND NOTICES.

ERRATUM.—In April issue, page 185, line 17th, for *neck* read *knee*.

NEW JERSEY STATE HOMŒOPATHIC MEDICAL SOCIETY meets at Continental Hotel, Newark, Tuesday, May 1st.

BELL ON DIARRHŒA.—A new edition of this justly celebrated work is now being prepared by Drs. Bell and Kimball. This will be welcome news to many physicians.

HOMŒOPATHY RECOGNIZED.—Dr. G. W. Sherbino has been recently appointed by District Judge Conner a member of the Board of Medical Examiners for the Forty-second Judicial District. A good appointment.

REMOVALS.—Dr. Julius Schmitt has removed his office and residence from 42 St. Paul Street, to 113 North Avenue, Rochester. Dr. Cornelia S. Simpson has removed from Bloomfield, New Jersey, to Hoosick Falls, New York. Dr. L. H. Bradley from Merced to Alameda, California. Dr. G. L. Long from Lone Pine to Merced, California. Dr. J. A. Gill, of the Homœopathic Medical College of Missouri, has gone to Twane, Mo. Dr. J. G. Gundlach removed some time ago from Texas to Spokane Falls, Washington Territory. Dr. G. is now so well pleased with his new home that he will settle there. Drs. F. Keller, Chas. S. Penfield, and C. D. Olmstead are also practicing at this place. Dr. Frederick Bruns has removed from Hotel Pelham, corner of Tremont and Boylston Streets, Boston, to 28 Cedar Street, Boston Highlands.

THE AMERICAN INSTITUTE.—Dr. Pemberton Dudley, General Secretary, sends out a preliminary notice of the coming meeting of the Institute at Niagara Falls. The sessions will be held at the International Hotel, beginning Monday evening, June 25th, and will close at noon on Friday, the 29th.

Full particulars as to hotel and railroad rates will be announced later. Any information in regard to the life, labors, etc., of any deceased member may be sent to the necrologist, Dr. H. D. Paine, 134 Madison Ave., New York city. All requests for information in regard to homœopathic statistics, etc., should be sent without delay to Dr. T. F. Smith, New York city.

THE ABUSE OF ACONITE.—Concerning the abuse of Aconite as a febrifuge, Dr. Farrington says: "* * * When fever is only a symptom, Aconite should not be given to control it. * * * You should no more attempt to lop off fever by the administration of Aconite than you would lop off one symptom in any disease. * * * Again a mistake is made in giving Aconite in typhoid fevers to diminish the pulse and control the temperature. * * * Aconite is seldom to be thought of in scarlatina. There may be exceptional cases where the fever is disproportionately severe and the *characteristic mental* symptoms *are present;* but nine cases out of ten would only be spoiled by use of Aconite. In inflammatory fevers Aconite must give way to other remedies unless this anxiety, restlessness, etc., be present."

WILMINGTON HOMŒOPATHIC HOSPITAL.—A philanthropic citizen of Wilmington, Delaware, Mr. J. Taylor Gause, President of the Harlan & Hollingsworth Company, has purchased the Hygiene Home in that city and given it, rent free, to the Homœopathic Free Hospital for a year, with the privilege of purchasing at the end of that time for the amount now paid by Mr. Gause, which is about $25,000. This action places the Homœopathic Hospital on a firm footing. Enough money has already been subscribed to carry it through its first year, and the hospital will be ready for patients by the last of November.

FAITH CURES.—While sound theology condemns modern miracles, it is impossible for pathologists and psycologists to treat them so cavalierly. In them are exhibited, in a more or less legitimate manner, the results of the action of the mind upon the bodily functions and particles. Hysteria is curable by these phenomena, since hysteria, after all, is only an unhealthy mastery of the body over the mind, and is cured by any stimulus to the imagination, which reverses the position, but at the same time the mind can produce actual morbid anatomical changes in the body, and as the experiments of French physicians in hypnotism tend to show, even favorable structural and functional alterations, Therefore there is no reason to doubt that faith-healing may have more positive results than we have been accustomed to allow it.—*British Medical Journal.*

TOO MUCH SURGERY.—At a meeting of the Philadelphia County Medical Society, Dr. John C. DaCosta said: Believing that many unnecessary operations are done, and that it is better practice to cure a woman and leave her with her organs intact, rather than by a brilliant operation to unsex her, without the certainty and with only the hope of relief, I took decided ground at the last meeting of this Society, in November, against the great number of abdominal sections now being made, including those made for "pain alone" (which is the subject for this evening's discussion), citing these cases to sustain my argument, viz.:

First. That a large proportion of the operations as done, and for the reasons alleged for operating, are unjustifiable.

Second. That the published reports of many cases said to be cured by operation are unreliable. For, though the operator may have been honest in thinking, when he made his report, that he had cured his patient, the after-history of the case sometimes showed failure.

Third. That the spaying of women for pain alone, and for many of the other supposed causes of trouble for which it is done, and in the miscellaneous way in which it is done, is unwarranted.—*From Medical and Surgical Reporter, January 14th,* 1888.

